# Role of Innate Immunity against Human Papillomavirus (HPV) Infections and Effect of Adjuvants in Promoting Specific Immune Response

**DOI:** 10.3390/v5112624

**Published:** 2013-10-28

**Authors:** Alfredo Amador-Molina, José Fernando Hernández-Valencia, Edmundo Lamoyi, Adriana Contreras-Paredes, Marcela Lizano

**Affiliations:** 1 Unidad de Investigación Biomédica en Cáncer, Instituto Nacional de Cancerología, Av. San Fernando No. 22, Col. Sección XVI, Tlalpan 14080, México; E-Mails: aamf17@hotmail.com (A.A.-M.); ferhv@hotmail.com (J.F.H.-V.); adrycont@yahoo.com.mx (A.C.-P.); 2 Instituto de Investigaciones Biomédicas, Universidad Nacional Autónoma de México, Apartado postal 70228, Ciudad Universitaria, Distrito Federal CP 04510, México; E-Mail: lamoyi@biomedicas.unam.mx

**Keywords:** human papillomavirus, dendritic cells, Langerhans cells, toll-like receptors, pro-inflammatory cytokines, natural killer cells, natural killer T cells, alpha-galactosylceramide

## Abstract

During the early stages of human papillomavirus (HPV) infections, the innate immune system creates a pro-inflammatory microenvironment by recruiting innate immune cells to eliminate the infected cells, initiating an effective acquired immune response. However, HPV exhibits a wide range of strategies for evading immune-surveillance, generating an anti-inflammatory microenvironment. The administration of new adjuvants, such as TLR (Toll-like receptors) agonists and alpha-galactosylceramide, has been demonstrated to reverse the anti-inflammatory microenvironment by down-regulating a number of adhesion molecules and chemo-attractants and activating keratinocytes, dendritic (DC), Langerhans (LC), natural killer (NK) or natural killer T (NKT) cells; thus, promoting a strong specific cytotoxic T cell response. Therefore, these adjuvants show promise for the treatment of HPV generated lesions and may be useful to elucidate the unknown roles of immune cells in the natural history of HPV infection. This review focuses on HPV immune evasion mechanisms and on the proposed response of the innate immune system, suggesting a role for the surrounding pro-inflammatory microenvironment and the NK and NKT cells in the clearance of HPV infections.

## 1. Introduction

Cervical cancer is the second most common cancer in women worldwide, and human papillomavirus (HPV) infection is the main risk factor for developing this disease [1]. More than 100 HPV types have been identified [2] and 38 of them can infect the anogenital tract. According to their oncogenic potential, HPVs are classified as high- (HR-HPV) or low-risk (LR-HPV), with the former being associated with anogenital cancer and the latter, with genital warts or epithelial lesions. HPV16 is the type that is most frequently found in cases of cervical cancer, followed by HPV18 [3,4].

HPV is a DNA virus with a circular genome of approximately 8000 bp that contains an early region, encoding the early viral proteins E6, E7, E8, E1, E2, E4 and E5, and a late region, encoding L1 and L2 proteins, which are components of the viral capsid. The long control region (LCR) is a non-encoding region involved in replication and viral transcription.

The expression of the viral proteins is associated with the cell differentiation program, and these proteins are therefore differentially expressed in the layers of the cervical epithelium [5]. The proteins that are first expressed are E1 and E2, which regulate viral replication and transcription. The formation of an E1-E2 complex is required for the stable binding of the E1 helicase to the LCR ori site [6]. E2 is a transcriptional regulator of early expressed HPV genes; when E2 binds to the four E2-binding-domains in the LCR it controls the transcriptional levels of E6 and E7 viral oncogenes.

The transformation step is not a common occurrence of an HPV infection, and only a small number of cervical lesions infected with high-risk HPV types evolve into cervical cancer [7]. Sometimes, for yet unknown reasons, the HPV genome integrates randomly into the host DNA. During this process, the HPV DNA often breaks at any position within the E1-E2 region. When E2 is lost, E6 and E7 become actively expressed, promoting cervical transformation [8,9,10].

The immune response plays an important role in clearing most of these infections, but some infections cannot be eliminated and persist for several years, becoming an additional risk factor [11].

During the early stages of an HPV infection, the host innate immune response becomes the first line of defense against the infection. Dendritic (DC), Langerhans (LC), natural killer (NK), natural killer T (NKT) cells and keratinocytes, among others, are important cells involved in promoting a good adaptive immune response against HPV infection and are the focus of this review. Most of these cell types can promote a cytokine-mediated pro-inflammatory process, which links the innate with the adaptive immune response. Moreover, NK cells are able to directly eliminate HPV infected cells [12].

However, HPV can evade the immune response, mainly through the action of E6 and E7 proteins. The viral mechanisms of immune evasion range from modulation of cytokines and chemo-attractant expression to alteration of antigen presentation, and down-regulation of IFN-pathways and adherence molecules [13]. Evasion of the immune response by HPV is critical for a successful infection.

Thus, stimulation of the innate immune response through strong adjuvants has turned out to be a promising therapeutic strategy for disrupting the evasion mechanisms of HPV and has been useful to understand the function of some innate immune cells during HPV infections.

## 2. Keratinocytes at the Initiation of HPV Infections

HPV infects keratinocytes of the basal layer of the cervical epithelium [14,15] and possibly stem cells [16,17]. As the main target of HPV, the keratinocyte plays an important role during the initiation of the HPV infection and subsequently becomes a link to promote an effective adaptive immune response. The keratinocytes are part of the innate immune defence system and have been considered as immune sentinels [18]. They can function as non-professional antigen presenting cells, and are able to induce the expression of T_H1_ and T_H2_ type cytokines and cytotoxic responses in CD4+ and CD8+ memory T cells, respectively [19]. Keratinocytes in female genital tracts express several Toll-like receptors (TLRs), located either on the cell surface (TLR-1, TLR-2, TLR-4, TLR-5 and TLR-6) or in the endosomes (TLR-3 and TLR-9) [20]. The TLRs are a family of immunological receptors that recognize pathogen-associated molecular patterns (PAMPs), their activation initiates signaling pathways that result in innate and adaptive immune responses. Endosomal TLRs play an important role in combating viral infections and in the recognition of viral nucleic acids; TLR-3 recognizes double-stranded RNA (dsRNA), TLR-7 and TLR-8 single-stranded RNA (ssRNA), and TLR-9 double stranded CpG-rich DNA. The activation of these receptors promotes the production of cytokines and creates a powerful pro-inflammatory environment [21,22,23], in particular, activation of TLR-9 in keratinocytes results in production of TNF-α, IL-8, CCL2, CCL20, CXCL9 and type 1 IFN [24,25]

**Figure 1:**
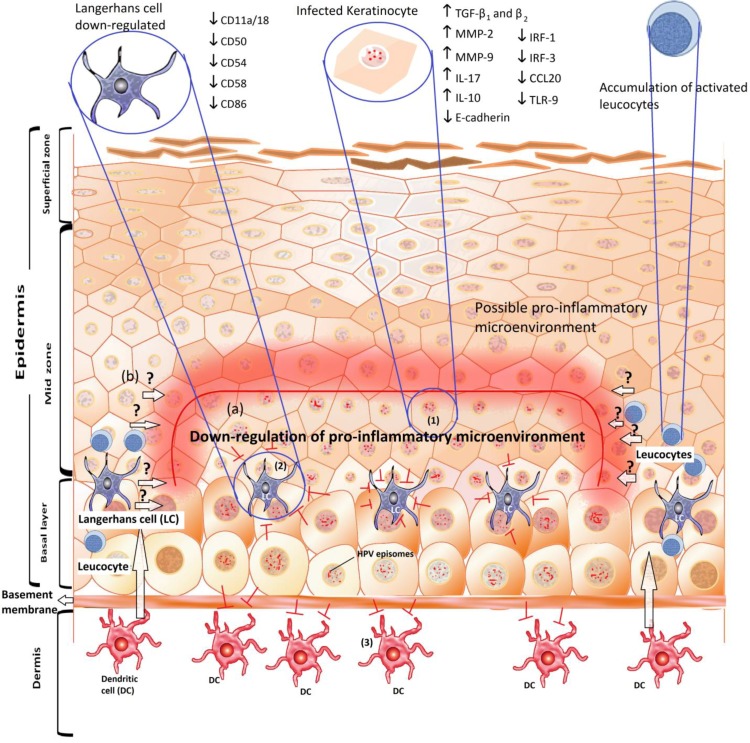
Schematic representation of the immunological microenvironment in a human papillomavirus (HPV) infection. (**a**) The predominant microenvironment induced by HPV promotes a down-regulation of antigen presentation, which triggers the following phenomena: (1) modulation of the cytokine-mediated inflammatory response of keratinocytes as the first line of defense against infection; (2) the inhibition of the activation and migration of Langerhans (LCs); and (3) evasion of the infiltration of dendritic cells (DCs) from the stroma. (**b**) The possible pro-inflammatory microenvironment in keratinocytes adjacent to the lesion. This microenvironment is characterized by down-regulation of the anti-inflammatory cytokine IL-10 and the presence of activated T-cells. The arrows with a question mark indicate an unknown process that could reverse the HPV-induced microenvironment.

HPV is able to modify cytokine levels as an immune evasion mechanism [26]. This strategy is mainly directed to down-regulate the pro-inflammatory response in cervical keratinocytes [18]. Figure 1 shows the up- and down-regulated cytokines that can be found in the microenvironment of an HPV infected tissue.

Interferons (IFNs) are components of the innate immune system that mediate intracellular protection against viruses through antiviral, anti-proliferative and immunostimulatory mechanisms [27]. The regression of HPV lesions could be related to an interferon response. However, HPV oncoproteins reduce the IFN and MCP1 secretion by the keratinocyte; HPV18-E7 can reduce IRF-1 expression in cervical tissue from transgenic mice expressing HPV18 E6/E7 [28], while HPV18-E6 can inhibit the phosphorylation of molecules involved in IFN signaling as Tyk2 kinase, STAT1 and STAT2, in cervical cancer cell lines [29]. Interestingly, HPV16 positive patients with pre-malignant lesions respond to IFN-α treatment more effectively when the levels of E7 transcripts are low.

Keratinocytes containing episomal copies of HR-HPV display a large number of deregulated genes involved in chemotactic and pro-inflammatory mechanisms; down-regulated genes are involved in an innate and adaptive immune response as well as KC differentiation [30]. These results emphasise the importance of the keratinocyte as an initiator of the immune response against HPV and as a link to the adaptive immune response.

## 3. Role of DCs in HPV Infections

The professional antigen presenting cells (APC) orchestrate a T-cell-inducing response, which has been correlated with good clinical prognosis [31]. DCs are APC that promote T-cell immune response through the capture and presentation of antigens [32]. Human DCs in skin comprise LC cells in epidermis and three subsets in dermis, characterized by the expression of CD1a+, CD14+, or CD141+ [33]. There are several functional and phenotypic differences between LCs and DCs in skin: LC express fewer TLRs including -1, -2, -3, -6, -10 and promote CD8+ T cell responses through IL-15 [34,35], whereas dermal CD14+ DCs express TLR-2, -4, -5, -6, -8, -10 [36] and produce IL-1α, TGF-β, IL-10, IL-12, GM-CSF, IL-6 and IL-8 [35,36]. To generate an effective immune cellular response, the epithelium should be communicated with LCs and DCs.

Immunosuppressive DC subsets have also been described in humans. Nevertheless, their role in HPV infections is not yet clear. The suppressive role of some DC subsets has been explained through the activation of regulatory T cells (T_reg_) [37]. A suppressive phenotype on murine DCs can also be conferred by immune-regulatory molecules as indoleamine 2,3-dioxygenase (IDO) 1; conventional and plasmacytoid dendritic cells expressing IDO1 mediate a potent T cell suppression that predominates over the T cell stimulatory properties of other DCs, promoting suppression of antitumor immune responses [38,39]. A skin graft of HPV16-E7 transgenic mice with infiltrating langerin^−ve^ dermal dendritic cells expressing IDO1 is not rejected in non-transgenic mice; but the inhibition of IDO1 activity promotes E7-skin graft rejection [40].

The immune-regulatory programmed death-1 (PD-1) molecule and its ligand (PD-L1) are molecules that can also confer a DC immunosuppressive phenotype. PD-1 and PDL-1 are both commonly expressed on lymphocytes; the interaction of PD-1 on T-cells with its ligand expressed on APC promotes T-cell functional exhaustion and anergy [41,42]. The DC-PD-L1+ population is more abundant in cervical cells from HR-HPV (+) patients without intraepithelial neoplasia than in HR-HPV (−) patients; therefore, DC-PD-L1+ subset is possibly associated with the down-regulation of T_H1_-type cytokines in HR-HPV (+) patients [43]. 

The complete characterization of DC subsets will be necessary to understand the role of APC in HPV immune evasion mechanisms as well as to identify the success or failure of future treatments through vaccines and/or new adjuvants.

The migration and adhesion of APC are essential mechanisms during the initiation of an immune response against HPV infection, but unfortunately for the host, HPV can modulate APC adhesion and migration [44,45]. Down-regulation of E-cadherin by E6 and E7 disrupts the adhesion of keratinocytes to LCs [44]. However, silencing of E7 oncogene in HPV16-infected keratinocytes has been shown to restore E-cadherin expression [46]. Silencing both E6 and E7 allows the re-expression of CCL20 in HPV-positive cell lines; CCL20 is an important chemokine involved in the infiltration of immature LC to the epithelium [45,47].

A decrease in LC numbers has been found in cervical intraepithelial neoplasia (CIN) and is associated with the severity of the lesion [48]. LCs in CIN show little or no expression of the adhesion/costimulatory molecules CD11a/18, CD50, CD54, CD58 and CD86, suggesting a poor antigen-presenting environment. On the other hand, the expression of HLA-DR, CD54 and CD58 in keratinocytes increases with disease severity, which together with the observed accumulation of activated leucocytes below the lesion, point to the development of a CIN-related but weak immune response since TNFα is down-regulated and IL-10 expression is increased [49]. Whether such a response causes spontaneous regression of the lesion or clearance of the infection is uncertain.

Escape from the immune-surveillance induced by HPV also impacts the number of LC. In α-, γ- and µ-HPV infections, the LC population decreases, in contrast to β-HPV infections [50]. Therefore, important effects of HPV infection on the development of a cervical neoplasia include interference of the keratinocyte response, a decrease in the number of LC and down-regulation of LC activation markers. Figure 1b illustrates a CIN-related immune response, with a possible pro-inflammatory microenvironment at the epithelium adjacent to the lesion.

## 4. Down-Regulation of Toll-Like Receptors by HPV and the Use of TLR Agonists to Improve Immunity

The main TLR in relation to double-stranded DNA virus infection is TLR-9. The expression of this TLR is down-regulated in keratinocytes expressing the HPV16 and HPV-18 E6 and E7 proteins, in HPV16-positive cervical cancer samples and in HPV positive cell lines. TLR-9 expression can be rescued by silencing of E6 and E7 using siRNAs [51].

**Figure 2:**
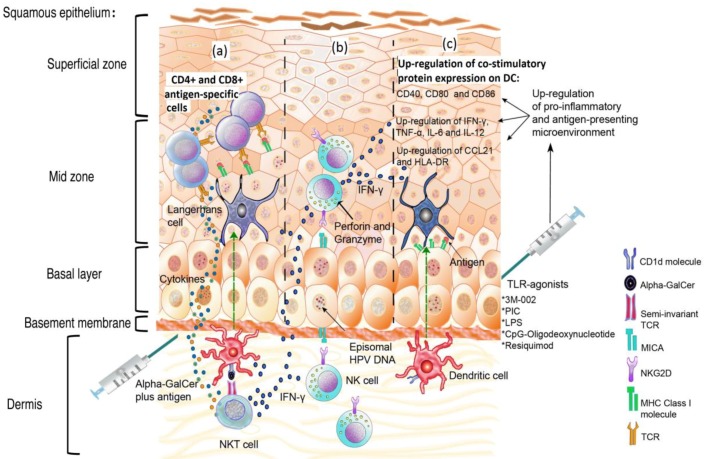
Schematic representation of the role of the innate immune response following adjuvant stimulation during HPV infection. (**a**) Stimulation of natural killer T (NKT) cells using α-GalCer, together with HPV-antigens, promotes CD4+ and CD8+ T cells antigen-specific responses and the rapid release of high levels of inflammatory cytokines, such as IFN-γ. (**b**) NK cells can kill HPV-infected cells, following their indirect activation through adjuvants such as α-GalCer and Toll-like receptor (TLR) agonists, via IFN-γ. (**c**) The induction of a pro-inflammatory response through TLR agonists rescues keratinocytes from the HPV-induced microenvironment to promote antigen presentation.

TLR-9 synthetic agonists have been used as a strategy against E7-expressing tumors in animal model systems. Figure 2a–c displays the proposed modulation of the innate immune response following adjuvant stimulation during HPV infection. In mice challenged with tumor cells constitutively expressing E6 and E7, the coinjection of recombinant E7 with ODN—oligodeoxinucleotide composed of unmethylated CpGs motifs (as depicted in Figure 2c)—induce a strong immunostimulatory effect resulting in a significant suppression of tumor formation, both prophylactically and therapeutically. The tumor protection appears to be driven by the activation of CD4+ and mostly by CD8+ T-cells, as demonstrated by *in vivo* T-cell subset depletion [52,53]. Table 1 describes different treatments that have been applied in order to improve immune response against HPV antigens.

Other TLR agonists that show non-canonical action against DNA virus infections also promote an efficient response against HPV proteins. Such agonists include 3M-002 (TLR-8 agonist) and resiquimod (TLR-8 and 7 agonist), which together with virus-like-particle VLP-L1-L2 or VLP-L1-L2-E7 (Table 1), are able to activate LCs, to induce the overexpression of chemokines and pro-inflammatory T_H1_ cytokines (MIP, IL-6, TNF-α, IL-12 and IL-8), to stimulate LC migration related to CCL21, and to induce a specific CD8+ T-cell response [54]. In contrast, the single antigenic stimulation with either of the HPV16 antigens is not sufficient to initiate an effective immune response and promote cytokine production [54]. A possible role of 3M-002 and resquimod in human HPV-infected tissue is depicted in Figure 2c. The Lipopolysaccharide TLR-4 agonist (LPS) and the polyinosinic acid-polycytidylic acid TLR-3 agonist (PIC), together with HPV11/E7 epitopes, can up-regulate CD40, CD80, CD86, CD83, HLA-DR, cytokines, as IL-12 and IFN-γ, in monocyte-derived dendritic cells (mdDC), and can also promote specific cytotoxic T lymphocyte response [55] (Table 1). A possible role of PIC and LPS in human HPV infected tissue is depicted in Figure 2c.

The ability to induce CD8+ T cells using TLR agonists is a useful finding, obtained from the study of adjuvants that aimed to promote a successful long-term immune response against intracellular pathogens. Therefore, the examination of TLR agonists against HPV proteins has become a promising field of inquiry, offering new possibilities for using adjuvants to promote a cellular response for the production of future HPV vaccines. TLR agonists could be useful in treatments intended to disrupt the anti-inflammatory microenvironment generated by E6-E7 HPV positive cells and the tolerance produced by HPV oncoproteins.

**Table 1:** Treatments used to improve immune response against HPV antigens.

Treatment	Effect	Model	Reference
CpG ODN (TLR-9 agonist) + E7 recombinant protein	Suppression of tumor formation.	Mouse	[52,53]
3M002 (TLR-8 agonist), resiquimod or (TLR-8 and 7 agonist) + VLP-L1-L2 or VLP-L1-L2-E7	Overexpression of chemokines and pro-inflammatory T_H1_ cytokines (MIP, IL-6, TNF-α, IL-12, IL-8). Stimulation of LC migration related to CCL21. Induction of specific CD8+ T cell response.	Human immune cells isolated from peripheral blood lymphocyte (PBL)	[54]
LPS (Lipopolysaccharide) TLR-4 agonist or polyinosinic acid-polycytidylic acid (PIC, TLR-3 agonist) + HPV11-E7 epitopes.	Up-regulation of CD40, CD80, CD86, CD83, HLA-DR, IL-12 and IFN-γ, in monocyte-derived dendritic cells (mdDC). Promotion of specific cytotoxic T lymphocyte response.	Human immune cells isolated from PBL	[55]
Live or inactivated *Listeria monocytogenes* or endotoxin.	Promote E7-specific T CD8+ cell immune response.	E7-Skin graft challenge.	[56]
Hydralazine and valproate	Decrease of soluble MICA and increase of susceptibility of target cells to NK attack.	NK cells isolated from PBL and tumor cells lines.	[57]
Short hairpin RNA (shRNA) plasmid targeting the IDO gene	Susceptibility to NK cell attack.	*In vitro* assays.	[58]
Gardasil HPV vaccine	Induction of protective antibodies. Increase NK cell population following immunization. Increase of the expression of NKG2D, NKp30, Nkp46 and ILT2 receptors.	Peripheral blood samples from vaccinated patients.	[26,59]
α-GalCer + DNA vaccine encoding the HPV16-E7 oncoprotein.	Increase of E7-specific CD8+ T cells and inhibition of tumor growth.	Mouse	[60]
β-GalCer	Inhibition of TC-1-tumor growth.	Mouse	[61]
B subunit of Shiga toxin coupled with ovalbumin or the E7 polypeptide + α-GalCer	Break tolerance generated against self Ag-elicited antiviral immunity	Mouse	[62]

## 5. The Pro-Inflammatory Response: Deregulation of the Link between the Innate and the Acquired Immune Responses

Some findings have suggested that HPV immune evasion mechanisms may act in the early stages of infection as a necessary mechanism for successful viral infection. The microenvironment of low-grade cervical lesions is predominantly anti-inflammatory, and is modified in a way that favors HPV infection. In cervical secretions obtained from low-grade squamous intraepithelial lesions (LSIL) from HPV-positive patients, higher levels of IL-10 are found compared to HPV-negative patients [63]. IL-10 is not always found in LSIL; nevertheless, there is evidence of over-expression of other anti-inflammatory cytokines, such as TGF-β1 and TGF-β2 [64]. 

Once the infection has been established, E6 expression leads to IL-17 up-regulation, which might constitute an important step for tumor development and progression, as demonstrated in E6-positive lung tumor cells [65]. IL-17 promotes angiogenesis and tumor growth [66], and as a component of the IL-17 signaling pathway, the IL-8, which is involved in angiogenesis and metastasis, is also up-regulated by E6 in adenocarcinoma lung cells. This IL-8 up-regulation is correlated with the expression of the MMP-2 and MMP-9 metalloproteinases, which are involved in angiogenic mechanisms [67] (Figure 1a). Modulation of the pro-inflammatory response may be an important step during HPV-carcinogenesis, a process that includes the establishment of the infection, persistence, progression, angiogenesis and metastasis.

The induction of a pro-inflammatory response can be used as a tool to break the tolerance induced by HPV [56]. The effectiveness of this approach has been demonstrated in models of E7-transgenic as well as non-transgenic mice, that when receiving an E7 transgenic skin graft were unable to respond to the E7 antigen. In these mice, stimulation with live or inactivated *Listeria monocytogenes* or endotoxin was sufficient to promote an E7-specific, CD8+ T cell immune response leading to the rejection of E7-grafts [56] (Table 1). Therefore, the successful initiation of pro-inflammatory signaling is important for developing new treatments to induce an effective immune response and disrupt the anti-inflammatory barrier triggered by HPV infections.

## 6. Natural Killer Cells: An Important Barrier against Cells Expressing HPV Antigens

NK cells represent an important barrier and a key component of the innate immune system. These cells have the capacity to recognize and kill virus-infected and transformed cells through two mechanisms: granule-dependent cytotoxicity; and the apoptosis pathway in the target cells [68] Nevertheless, tumor cells have developed mechanisms to evade being attacked by NK cells, and viruses such as HPV display intrinsic strategies for preventing infected cells from being easily eliminated by NK cells.

NK cell activity is tightly regulated through a balance between inhibitory and activating receptors [69]. However, deregulation of these receptors is common in cancer and HPV infections. NKp30 and NKp46 receptors are found at low levels in NK cells from patients with cervical cancer and precursor lesions, which is correlated with low cytotoxic activity of NK cells [70].

Another important receptor in NK cells that is related to cytotoxicity is NKG2D. This receptor is involved in cell lysis through the interaction with the major histocompatibility complex class I-related chain A (MICA) proteins. Both NKG2D and MICA are modulated in the presence of HPV-infection. Furthermore, the levels of free-MICA in serum are increased in association with cervical cancer progression, which suggests that a significant factor that contributes to HPV persistence or tumor progression could be the presence of soluble MICA in the serum [71]. In a NK cell line (NKL), it was found that the NKG2D receptor was down-regulated when the NKL was co-cultured with cervical cancer cell lines HeLa, SiHa, or C33A, but not with immortalized keratinocytes HaCaT [72]. This down-regulation of NKG2D in the NKL was associated with a reduced cytotoxic activity after contact with the HPV-positive cancer cell lines (HeLa, SiHa), but not after contact with HPV-negative cell line C33, or non-tumorigenic HaCat cell line [72].

Certain drugs are able to reduce MICA plasmatic levels, allowing the attack of NK cell to target cells that express MICA. For example, the administration of hydralazine and valproate can increase the expression of MICA and MICB ligands in the CaSki cervical cancer cell line and reduce their shedding to the supernatant, allowing NK attack; while cells without hydralazine and valproate are not susceptible to NK attack [57] (Table 1).

Several studies have evaluated the association of HLA class I and class II genes with susceptibility to cervical cancer [73,74], and their findings support the association of HLA polymorphisms with the risk of cervical neoplasia.

HPV can affect NK cells through different target molecules. Carcinoembryogenic antigen-related cell adhesion molecule 1 (CEA-CAM1) mediates NK cytotoxicity. CEA-CAM1 expression is increased in patients with high-grade squamous intraepithelial lesions (HSIL) in contrast to what is observed in LSIL, in which CEA-CAM1 is undetectable or present at a low level [75].

HPV can also evade the cytotoxic mechanisms of NK cells through the alteration of immunosuppressive enzyme indoleamine-2,3-dioxygenase (IDO), and IDO expression is correlated with the escape of tumor cells from immune surveillance [76]. The absence of IDO is related to increased NK cell activity. CaSki cells transfected with a short hairpin RNA (shRNA) plasmid targeting the IDO gene (shIDO) are more susceptible to NK cell attack *in vitro* than control cells (CaSki/Mock). Additionally, an *in vivo* assay performed in BALB/c nude mice revealed a greater accumulation of NK cells in the stroma of CaSki/shIDO formed tumors than in control subcutaneous tumors. Moreover, low level of IDO increases the susceptibility of cervical cancer cells to NK cells, suggesting that IDO-targeted shRNAs may represent an effective molecular targeted therapy for cervical cancer [58] (Table 1).

Although some of the mechanisms employed by HPV to avoid NK cell activity are known, the role of NK cells in the natural history of infection is not entirely clear. Moreover, there are few studies addressing the effects of adjuvants or drugs that might increase the number of NK cells and their cytotoxic activity against HPV infected cells during the early stages of infection or against HPV-positive tumor cells, in the later stages. As shown in Figure 2, some adjuvants may indirectly activate NK cells via IFN-γ (Figure 2).

The currently available Gardasil HPV vaccine can increase the NK cell population following immunization, which is associated with increased expression of NKG2D, NKp30, Nkp46 and ILT2 receptors in NK cells, suggesting the contribution of other pathways, besides the increase in neutralizing antibodies, involved in vaccine effectiveness [59] (Table 1). A detailed understanding of NK cell responses could lead to the generation of new and more effective immunotherapies against HPV-related cancer and/or early infections.

## 7. The Promising Role of NKT Cells in Controlling HPV Infection

Invariant or type 1 natural killer T cells (iNKT) are a group of T lymphocytes defined as CD1d1-restricted T cells that express a semi-invariant αβ T cell antigen receptor (TCR) and surface antigens typically associated with natural killer cells such as CD161 in humans [77,78,79].

The TCR found on type 1 NKT cells recognize glycolipid antigens presented by the MHC class I-related glycoprotein CD1d, which are expressed abundantly on antigen-presenting cells and other cell types. There are two ways in which iNKT cells are activated: directly, via engagement of the invariant TCR with glycolipid antigens and CD1d molecules, and indirectly, via activated antigen-presenting cells. After stimulation, iNKT cells rapidly secrete large amounts of various cytokines including IFN-γ, TNF, IL-4, IL-10 and IL-3, among others [80,81].

Due to the reciprocal activation of NKT cells and DC, synthetic NKT ligands constitute promising new vaccine adjuvants [82]. One of the best-studied antigens is α-galactosylceramide (α-GalCer), a molecule originally isolated from sponge *Agelas mauritianus*; closely related glycolipids are found in a broad range of microorganisms including bacteria of the genera *Novosphingobium*, *Borrelia* and *Streptococcus* [83,84]. A potent variant of α-GalCer called KRN7000 can enhance the immune system response against tumors, viruses, bacteria and parasites [83,85].

While the α-GalCer is one molecule that has been demonstrated to strongly stimulate NKT cells, there are endogenous antigens that can also stimulate NKT cells [86]; however, their activation capacity is lower than that of α-GalCer. Although HPV does not have NKT-stimulating glycolipids, whether HPV infection can modify the profile of endogenous glycolipids which can be presented to iNKT cells is unclear.

NKT cells have long been implicated in tumor immunity. In a murine model of adoptive immunotherapy using an established tumor expressing E7 from HPV16 (TC-1), NKT cells were necessary to inhibit early but not late tumor growth [87].

The role of NKT cells in the spontaneous regression of HPV lesions is uncertain. Results from immunodeficient or immunocompetent individuals suggest that the immune system has a significant role in the success or failure of spontaneous clearance [88]. However, the level of circulating NKT cells is not associated with the severity of infection or progression to cervical cancer [89]. Since HPV is a local infection, understanding the contribution of NKT cells in infected cervical tissue is necessary to identify the determinants of HPV clearance.

Despite the unclear role of NKT cells in HPV infections, there is evidence of immune evasion mechanisms that have been developed by HPV to avoid NKT cell activity. Some of these mechanisms are related to decreased CD1d expression, as observed *in vivo* in cervical HPV-infected tissues and *in vitro* in the C33A/CD1d+ and Vag/CD1d+ cell lines, which are stably transfected with HPV6 E5 and HPV16 E5, respectively. In these cell lines, the E5 protein targets CD1d to the cytosolic proteolytic pathway by inhibiting calnexin-related CD1d trafficking [90]. Thus, reduced CD1d expression may represent a strategy for HPV-infected cells to evade protective immunological surveillance during early stages of infection.

Research on α-GalCer has made an important contribution to the understanding of the role of NKT cells during HPV-infection (as depicted in Figure 2). In a mouse model, with TC-1 tumor cells (expressing E6 and E7), the number of E7-specific CD8+ T-cells was found to be increased when α-GalCer was administered as an adjuvant in addition to DNA vaccine encoding the HPV16 E7 oncoprotein; in addition, this treatment generated potent anti TC-1 tumor effects [60] (Table 1). Administration of β-GalCer(C12) without HPV antigen was also able to inhibit the growth of TC-1 at the early stages of tumor progression [61] (Table 1).

As depicted in Figure 2a,b α-GalCer coadministered with several immunogens such as proteins, recombinant virus and tumor cells has been shown to augment the level of antigen-specific CD8 T cell response [91,92,93]. Administration of the B subunit of Shiga toxin coupled with ovalbumin or the E7 polypeptide plus α-GalCer results in a powerful CD8 response, breaking tolerance generated against self-antigen and protection against a challenge with OVA-expressing vaccinia virus; the effect was not observed with other adjuvants such as TLR-9 or TLR-3 agonists, or IFN-α [62] (Table 1). Treatment of antigen-activated CD8+ T cells with α-GalCer before adoptive transfer to tumor-bearing mice resulted in increased numbers of antigen-specific CD8+ T cells and cytotoxic activity; this effect involved iNKT and DC cells [94]. These results demonstrate the potential of NKT ligands to be used as therapeutic molecules for treating HPV-associated cancers.

Recently, immunosuppressive functions of NKT cells in transgenic mouse models expressing the HPV16 E7 protein in epidermal keratinocytes have been described [95,96]. CD1d-restricted NKT cells infiltrating E7-positive skin grafts inhibit their rejection, through the secretion of IFN-γ [95]. In addition, NKT cells from the lymph nodes draining the skin graft were capable of suppressing CD8 T cell proliferation, cytokine production and cytotoxic activity [96].

There are currently few available reports addressing the role of NKT cells in precancerous and cervical cancer lesions and their impact on the microenvironment surrounding HPV-infected cervical tissue. Understanding the role of NKT cells in cervical cancer will be useful for the design of alternative ways for immunotherapy.

## 8. Perspectives

Despite the great efforts exerted in HPV-vaccination programs, cervical cancer still represents the second most common cancer in women. Therefore, the mechanisms involved in HPV clearance and HPV immune evasion should be settled.

Innate immunity is the first barrier associated with HPV clearance through promoting humoral or cellular immune responses. The study of new molecules that stimulate the innate immune response, mainly through adjuvants, represents a new possibility to understand the mechanisms of innate immune evasion induced by HPV and the unclear role of the microenvironment surrounding an HPV infection. Such research could aid in the identification of new targets and the design of efficient therapies for treating HPV infections, where the complexity of tumor immunosuppressive mechanisms should be considered.

Recent studies using TLR agonists and α-GalCer adjuvants have shed light on the unclear roles of the surrounding environment and NKT cells, respectively, during HPV infections. However, exploring new adjuvants, or other molecules such as shRNAs targeting immunosuppressive molecules, will be necessary to improve our understanding of the role of NK cells in HPV infections.
